# Hydrogen Sulfide Signaling Axis as a Target for Prostate Cancer Therapeutics

**DOI:** 10.1155/2016/8108549

**Published:** 2016-02-25

**Authors:** Mingzhe Liu, Lingyun Wu, Sabine Montaut, Guangdong Yang

**Affiliations:** ^1^Cardiovascular and Metabolic Research Unit, Lakehead University, Thunder Bay, ON, Canada P7B 5E1; ^2^Department of Health Sciences, Lakehead University, Thunder Bay, ON, Canada P7B 5E1; ^3^Department of Chemistry and Biochemistry, Laurentian University, Sudbury, ON, Canada P3E 2C6

## Abstract

Hydrogen sulfide (H_2_S) was originally considered toxic at elevated levels; however just in the past decade H_2_S has been proposed to be an important gasotransmitter with various physiological and pathophysiological roles in the body. H_2_S can be generated endogenously from L-cysteine by multiple enzymes, including cystathionine gamma-lyase, cystathionine beta-synthase, and 3-mercaptopyruvate sulfurtransferase in combination with cysteine aminotransferase. Prostate cancer is a major health concern and no effective treatment for prostate cancers is available. H_2_S has been shown to inhibit cell survival of androgen-independent, androgen-dependent, and antiandrogen-resistant prostate cancer cells through different mechanisms. Various H_2_S-releasing compounds, including sulfide salts, diallyl disulfide, diallyl trisulfide, sulforaphane, and other polysulfides, also have been shown to inhibit prostate cancer growth and metastasis. The expression of H_2_S-producing enzyme was reduced in both human prostate cancer tissues and prostate cancer cells. Androgen receptor (AR) signaling is indispensable for the development of castration resistant prostate cancer, and H_2_S was shown to inhibit AR transactivation and contributes to antiandrogen-resistant status. In this review, we summarized the current knowledge of H_2_S signaling in prostate cancer and described the molecular alterations, which may bring this gasotransmitter into the clinic in the near future for developing novel pharmacological and therapeutic interventions for prostate cancer.

## 1. Introduction

Hydrogen sulfide (H_2_S) is a colorless, flammable gas with the characteristic odor of rotten eggs. H_2_S is traditionally considered as a toxic environmental pollutant with little or no physiological significance. The mechanism of H_2_S toxicity is thought to bind and inhibit mitochondrial cytochrome *c* oxidase, which is involved in cellular oxidative processes and energy production [1]. The inhibition of cytochrome *c* oxidase blocks the electron transport chain, decreases ATP production, and finally induces cell death. However, just in the last decade, H_2_S is acknowledged to be one important gasotransmitter, influencing plentiful physiological and pathological processes [2–6]. In 1998, three Nobel winners in Medicine and Physiology, Drs. Robert F. Furchgott, Louis J. Ignarro, and Ferid Murad, discovered that nitric oxide (NO) is an endothelial-derived relaxing factor and acts as a signaling molecule in the cardiovascular system. We now know that NO is the first identified gasotransmitter. Similar to NO, H_2_S possesses all the criteria to qualify as a gasotransmitter [4, 6]. First, H_2_S is a small gas with the simple molecular structure of two hydrogen atoms and one sulfide atom. Secondly, H_2_S has higher lipid solubility and can penetrate easily through cell membranes without using any specific transporter/receptor. Thirdly, H_2_S not only is found from the environment, but also can be endogenously generated in almost all organs and cells by specific enzymes through reverse-transsulfuration pathway. Fourthly, H_2_S generates various functions at physiologically relevant concentrations by targeting at specific cellular and molecular sites, which can be mimicked by exogenously applied H_2_S donors.

H_2_S is involved in an array of cellular signals regulating cardiac, neurological, and respiratory functions, as well as cellular metabolism and survival [5, 7]. Many diseases including cardiovascular diseases, neurological diseases, shock, sepsis, metabolic disorders, and cancers have been linked to abnormal endogenous H_2_S functions and metabolism [4]. Although H_2_S has numerous physiological functions, the actual levels of H_2_S present in biological tissues and fluids are not really known. The past studies have shown that the concentration of H_2_S in circulation ranged from nanomolar to micromolar one depending on different detection methods [2]. H_2_S usually provides cytoprotective effects at very low concentrations but is cytotoxic at higher concentrations via free radical and oxidant generation, glutathione (GSH) depletion, and initiation of proapoptotic gene expression [8]. It is proposed that H_2_S mediates all these cellular functions through protein posttranslational modifications. H_2_S can modify cysteine residues of the proteins and bind with the sulfhydryl group of cysteine forming persulhydral group (–SSH), termed as protein* S*-sulfhydration [9–13]. It is predicted that* S*-sulfhydration changes protein structure and alters protein activity and functions. The modified cysteine residue is highly reactive and usually increases the catalytic activity of targeted proteins [14].

## 2. H_**2**_S Biosynthesis and Metabolism

**Figure 1:**
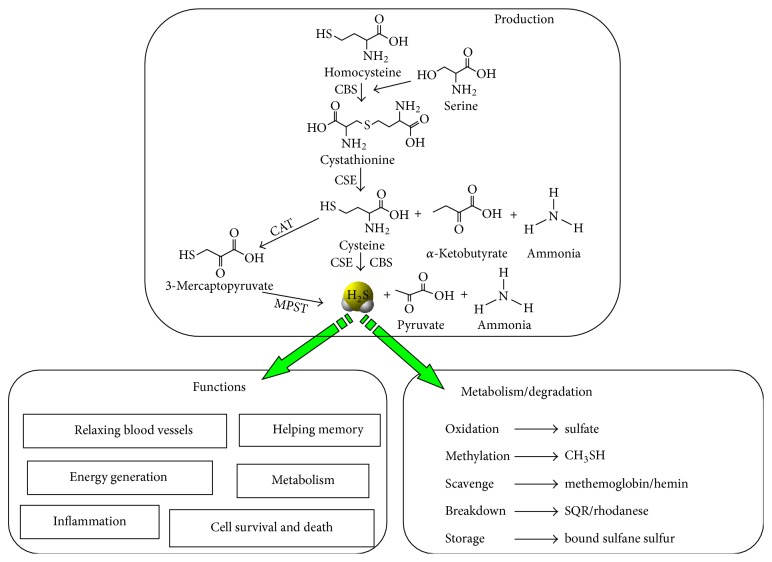
H_2_S biosynthesis, functions, and metabolism. So let us first look at how H_2_S is endogenously produced. In mammalian cells, H_2_S can be endogenously produced through the transsulfuration pathway. With cysteine as the main substrate, CBS or CSE, which we called cystathionine gamma-lyase or cystathionine beta-synthase, can catalyze cysteine into H_2_S and other products. The expression of CBS and CSE is tissue-specific, CBS is mostly expressed in brain, and CSE is in the cardiovascular system and other big tissues, such as liver and kidney. The half-life of H_2_S inside the cells is very fast; it is estimated in several seconds and can be quickly oxidized, scavenged, or broken down by different ways. Now it has been widely recognized that H_2_S plays very important physiological role in the whole body and also cells; for example, it can relax blood vessel and acts as an endothelial deprived hyperpolarizing factor, can enhance long term potentiation and help memory, and can control energy generation and regulate metabolism, inflammation; more importantly, H_2_S is required for cell fate decision; that is the topic we are going to talk about in the following.

Now it is recognized that endogenous H_2_S generation is from enzymatic and nonenzymatic pathways [4, 5]. At least 3 enzymes are responsible for endogenous H_2_S generation in mammalian cells, including two cytosolic pyridoxal-5′-phosphate-dependent enzymes, cystathionine beta-synthase (CBS, EC 4.2.1.22) and cystathionine gamma-lyase (CSE, EC 4.4.1.1), and a mitochondrial enzyme 3-mercaptopyruvate sulfurtransferase (MPST, EC 2.8.1.2) (Figure 1) [5, 8]. All these three enzymes use sulfur-containing amino acids L-cysteine as substrate to generate H_2_S. CBS catalyzes the condensation of serine and homocysteine to form cystathionine, and CSE then cleaves the C-*γ*-S bond of cystathionine to yield cysteine, *α*-ketobutyrate, and ammonia (NH_3_). Moreover, both CBS and CSE can use cysteine as substrate to produce H_2_S, and pyruvate and NH_3_ are two other byproducts. Cysteine aminotransferase (CAT) provides 3-mercaptopyruvate from cysteine for MPST to produce H_2_S. These enzymes are critical for the maintenance of H_2_S homeostasis by precisely regulating H_2_S levels in tissues [8]. It is worth noting that MPST functions more efficiently at very high pH. At physiological condition, the contribution of MPST to endogenous H_2_S production is negligible in comparison with CBS and CSE [15]. The expression of these 3 enzymes in the body is tissue-specific. They can be all expressed in one organ, or only one of them is expressed in the specific organ. In cardiovascular system, CSE probably is the major H_2_S-producing gene, helps vascular tone, and regulates blood pressure. In the brain and peripheral nervous system, CBS and MPST are the major H_2_S-producing enzymes, help brain for neuromodulation, and stimulate memory. In pancreas, both CBS and CSE can be expressed, but only CSE acts as the critical enzyme to produce H_2_S, regulating insulin release and cell survival of insulin-secreting beta cells. In lungs, so far only CBS and CSE are showed to be expressed, and H_2_S regulates airway contraction and delays asthma development. In intestine, H_2_S is of bacterial origin or also is produced from both CBS and CSE, helping inflammation and pain. In the large organ, for example, liver and kidney, all three enzymes are expressed, and H_2_S can be vastly produced by these two tissues [2, 3].

H_2_S can also be produced endogenously through nonenzymatic pathways and elemental sulfur, including thiosulfate, thiocysteine, and other molecules in the blood, which can be reduced to H_2_S through the glycolytic pathway (Figure 1) [5]. Another important source of H_2_S is from the H_2_S-producing bacteria existing in the intestinal system. The concentration of H_2_S inside the cells is accurately regulated to maintain the proper physiological function of H_2_S [7]. H_2_S can be quickly and spontaneously oxidized to thiosulfate and then to sulfite or sulfate in the presence of oxygen and Fe^3+^ iron in mitochondria. Recent reports showed that H_2_S also can be oxidized to polysulfides (H_2_S*n*), which are thought to be more stable than H_2_S and act as more potent signaling molecules [8]. In cytosol, H_2_S interacts with various proteins in the blood, including metalloproteins, disulfide-containing proteins, and thio-*S*-methyltransferase, forming methyl sulfides, while methylation of H_2_S is much slower than mitochondrial oxidation [5].

## 3. Expression of H_**2**_S-Generating Enzymes in Prostate Tissues

In 2012, Guo et al. thoroughly analyzed the expressions of H_2_S-generating genes in human prostatic tissue (epithelial and stroma cells) and different prostatic normal, benign, and cancer cell lines [16]. The prostatic tissue stromal compartments and stroma cell WPMY-1 presented middle to strong signals of CSE. The protein levels of CBS and CSE are greatest in the androgen-dependent prostate cancer cell LNCaP in comparison with all other cells. In LNCaP cells, both CBS and CSE are located in the cytoplasm as evidenced by immunostaining, and the CBS/CSE activities parallel the CBS/CSE protein levels [16]. In contrast, CBS and CSE are hardly detected in the normal prostatic peripheral zone epithelial cell line RWPE-1. Gai et al. further demonstrated that not only CBS and CSE but also MPST is present in human prostate tissue, and CSE is expressed at much higher level in comparison with CBS and MPST [17]. In contrast, Zhao et al. found that MPST is not expressed in both human prostate adenocarcinoma and normal prostate tissues [18]. The difference may be due to the detection method and antibody resource. Furthermore, Zhao et al. provided evidence that the expression of CSE but not CBS is significantly reduced in prostate cancer tissue when compared with normal prostate tissues [18]. CSE expression is also lower in antiandrogen-resistant prostate cancer cells in comparison with their parental LNCaP cells, whereas the expression of CBS is similar between these two types of cells. Pei et al. confirmed that both CSE and CBS are expressed in mouse prostate tissues, in both androgen-dependent and androgen-independent prostate cancer cells (LNCaP and PC-3) [19]. Both CBS and CSE use cysteine as substrate to produce H_2_S; however the contribution of CBS and CSE to H_2_S production in prostate tissue is quite different. Complete removal of CSE gene in mice eliminated H_2_S production by more than 80% in prostate tissues in comparison with that from wild-type mice, indicating that CSE but not CBS acts as a major H_2_S-producing enzyme in prostate.

## 4. Altered Sulfide Metabolism in Patients with Prostate Cancer

Prostate cancer is the most invasive and frequently occurring cancer among men with nearly a million new cases diagnosed worldwide annually [20]. Prostate cancer has approximately a sixfold higher incidence in Western than in non-Western countries. Prostate cancer arises from malignant transformation of prostate cells, and prostate cancer cells have the potential for invasion of neighboring organs and form metastases mostly in lymph nodes and bone [21]. Androgen ablation therapy and radical prostatectomy are the main treatment options for early stage prostate cancer. However in the final stage prostate cancer progresses to a castration resistant state that is highly aggressive, metastatic, and resistant to chemotherapy and finally causes the death of patient, which accounts for approximately 30,000 deaths in the US in 2014 [22]. Development of novel diagnosis and preventive interventions are urgent to reduce morbidity, mortality, and healthcare cost associated with this tumor. New markers of this aggressive disease are also critically needed for clinical decision.

Several lines of evidence recently demonstrated that altered sulfide metabolism is involved in patients with prostate cancer [23–25]. Mitochondria can oxidize H_2_S to thiosulfate and then to sulfite, which is excreted by the kidney in urine, so thiosulfate is a naturally occurring metabolic product from H_2_S. The concentration of thiosulfate will be increased in urine when people are exposed to H_2_S or if H_2_S metabolism is disrupted inside the body. Chwatko et al. recently compared the thiosulfate level in the urine samples from 166 prostate cancer patients, 42 benign prostatic hyperplasia cases, and 20 healthy people [26]. Interestingly, they found that the urinal thiosulfate level in prostate cancer patients is almost 50 times higher than in the control groups and 5 times higher than in the benign prostatic hyperplasia group, suggesting an impaired H_2_S metabolism in prostate cancer. Furthermore, Chwatko et al. observed that the level of thiosulfate is positively related with prostate tumor volume but not tumor stage and grade [26]. It is paradoxical that the level of thiosulfate does not correlate with serum prostate-specific antigen (PSA) level. It is not clear how thiosulfate level is higher in the urine samples from prostate cancer patients. Several enzymes, including thiosulfate/cyanide sulfur transferase (TST, rhodanese) and sulfite oxidase, are involved in H_2_S oxidation to thiosulfate [5]. Further analysis on the change of these enzymes will provide more clues on the altered thiosulfate level in prostate cancer.

In addition, Kimura et al. observed that the products of methionine catabolism are correlated with prostate cancer progression status [27]. Cysteine, the substrate required for all 3 enzymes to generate H_2_S, is significantly elevated in the urine samples from biochemically recurrent prostate cancer patients compared to those who remained recurrence-free five years following prostatectomy. Along with cysteine, homocysteine and cystathionine are also significantly higher in the biochemically recurrent patients, suggesting that cysteine, cystathionine, and homocysteine can act as independent predictors of recurrence-free survival for prostate cancer patients. In contrast, the concentration of cysteine is reported to be significantly lower in plasma as a result of prostate tumor progression in nude mice implanted with human prostate cancer cells [28, 29]. Further studies need to be clarified on the altered sulfide metabolism in patients with prostate cancer.

Multiple studies also showed that the other products from sulfide amino acid metabolism are higher in prostate cancer patient. Sarcosine (*N*-methylglycine), a product of methionine catabolism, is reported to be higher in the urine of patients with metastatic prostate disease and is also higher in tissues from localized prostate cancer than in normal tissue [30]. Therefore, urinary sarcosine can also be used as a possible marker for metastatic prostate cancer.

## 5. H_**2**_S Inhibition of Prostate Cancer Cell Growth

**Figure 2:**
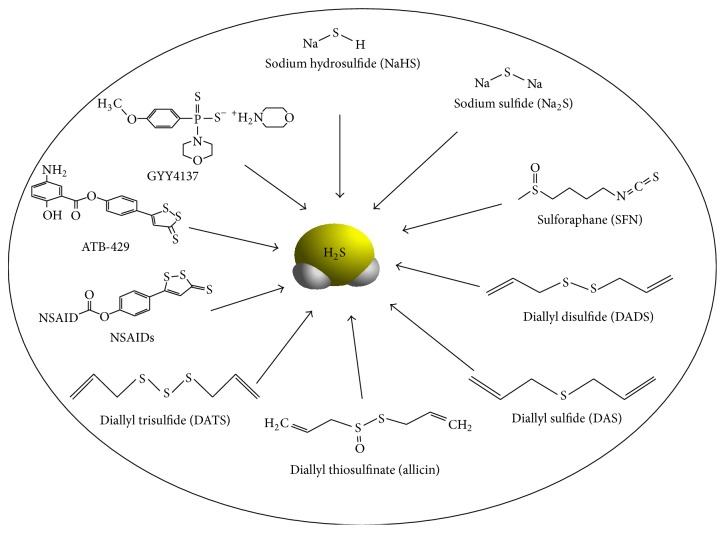
H_2_S-releasing donors.

The functional importance of H_2_S in the biology of the prostate cancer cells is recently recognized [31, 32]. Epidemiological, clinical, and laboratory studies have shown that H_2_S and/or sulfide-containing compounds inhibit the survival of prostate cancer cells* in vitro* and* in vivo* (Figure 2). An increased intake of garlic and cruciferous vegetables has long been associated with a reduced risk in the occurrence and progression of prostate cancer [33–35]. Garlic contains different sulfur-containing compounds (Figure 2), including diallyl disulfide (DADS), diallyl trisulfide (DATS), allicin, and allyl-methyl-thiosulfinate, which are useful organic sources of H_2_S* via* reactions involving alliinase-mediated enzymatic conversion of* S*-alk(en)yl-L-cysteine sulfoxide to alkyl alkane thiosulfinates, followed by instant decomposition of these byproducts [36–39]. Benavides et al. also reported that garlic sulfur-containing compounds are able to release H_2_S with a relatively slow mechanism in the presence of endogenous thiols, such as GSH [40]. In addition, Bhuiyan et al. provided evidence showing that TST catalyzes garlic extracts to release H_2_S* in vitro* in the presence of reduced thioredoxin [36]. Cruciferous vegetables uniquely contain a group of sulfur-containing compounds known as isothiocyanates, which can release H_2_S under specific conditions. Sulforaphane (SFN) (Figure 2) is one of the principle isothiocyanates which prevents or delays tumor development in a variety of animal models of prostate cancers [41, 42].

### 5.1. Sulfide Salts

NaHS is a well-used H_2_S donor, which can cause rapid H_2_S release (Figure 2). In physiological saline, NaHS dissociates into Na^+^ and HS^−^, and then HS^−^ associates with H^+^ to form H_2_S, and about one-third of the H_2_S exists in the undissociated form. Pei et al. first observed that NaHS at 50–200 *μ*M significantly decreases cell viability of PC-3 cells, an androgen-unresponsive metastatic cell line [19]. Blockage of the phosphorylation of both p38 MAPK and JNK reversed the inhibitory effects of NaHS on PC-3 cell viability. By using the same cell line, CSE overexpression enhanced H_2_S production and inhibited cell viability in PC-3 cells. This occurred also in androgen-independent prostate cancer cell line. Exogenously applied NaHS at 30 *μ*M significantly suppressed cell viability in both androgen responsive cells and antiandrogen-resistant cells in the presence or absence of R1881 [11]. Remarkably, in comparison with young mice, CSE expression and H_2_S production in prostate tissue from older mice were significantly reduced, accompanied by an increased cell proliferation evidenced by an increased expression of PCNA and cyclin D1. The authors indicated that CSE/H_2_S system may be essential for maintaining the balance of age-linked cell growth in prostate tissues.

In addition, Duan et al. investigated the inhibitory effects of sulfur on prostate tumor growth* in vivo* [43]. The* nude* mice were inoculated with prostate cancer cells (22Rv1 and DU-145) following feeding with 0.62 g/day sulfur-milk powder for 22 days, while the control mice inoculated with prostate cancer cells were only provided with milk powder. Serum H_2_S level in the sulfur-treated mice was significantly increased. The rate of growth of tumors in sulfur-treated mice was markedly reduced when compared with that of the control group. The prostate cancer cells separated from the sulfur-treated xenograft tumors formed much lower clones than that of the control tumors, indicating that the clonogenicity of 22Rv1 or DU-145 prostate cancer cells is significantly decreased by sulfur. Interestingly, as early as forty years ago, clinical practice had showed that treatment with H_2_S water improves blood supply to the prostate gland in patients with chronic prostatitis, pointing to the beneficial role of H_2_S in prostate tissue under pathological condition [44].

### 5.2. DADS

DADS (Figure 2) is one of the principal organosulfur compounds from garlic and a few other* Allium* plants [45, 46]. Arunkumar et al. proved that DADS at 10–50 *μ*M inhibits cell survival and induces cell apoptosis of androgen-independent prostate cancer cells (PC-3) in a dose-dependent manner [47, 48]. DADS was found to downregulate the expression of insulin-like growth factor signaling system, which subsequently leads to inhibition of Akt phosphorylation and the expressions of cyclin D1, NF*κ*B, and antiapoptotic Bcl-2 protein, but increases proapoptotic signaling proteins (Bad and Bax), thereby inhibiting cell cycle progression and survival. The same group further demonstrated that DADS provides chemopreventive activity in rat prostate carcinogenesis [49], which was induced by injecting the rats with testosterone and* N*-methyl* N*-nitrosourea (MNU) throughout the experimental period.

Chen et al. found that DADS induces cell death in PC-3 cells by stimulating Ca^2+^ release from endoplasmic reticulum in a phospholipase C-independent manner and also causing Ca^2+^ influx via phospholipase A2-dependent manner [50]. Many other studies also confirmed that DADS suppressed the proliferation of prostate cancer cells through cell cycle arrest and apoptosis [51–53]. It is clear that DADS may be used for further drug discovery approach in the prostate cancer therapy.

### 5.3. DATS

Similar to DADS, DATS (Figure 2) is also a natural product with a pungent odor and volatility when isolated from garlic and has been shown to have anti-prostate cancer activity both* in vitro* and* in vivo* [54–58]. DATS significantly induces cell death of prostate cancer cells (PC-3) but not of noncancerous human prostate epithelial PNT1A cells. DATS stimulated more ROS formation, ferritin degradation, inactivation of Akt, and activation of ERK1/2 in PC-3 cells in comparison with PNT1A cells, which may explain the higher sensitivity of prostate cancer cells to the cytotoxic effects of DATS [54, 59]. Sielicka-Dudzin et al. showed that DATS induces cell death of prostate cancer cells (PC-3) via JNK1-dependent ROS formation and Itch-dependent ferritin degradation, while DATS-induced cell cycle arrest in DU145 cells is associated with delayed nuclear translocation of cyclin-dependent kinase 1 [60, 61]. Chen et al. further confirmed that DATS and its derivatives, including dibutenyl trisulfide (DBTS), bis(2-methylallyl) trisulfide (2-M-DATS), dipentenyl trisulfide (DPTS), bis(3-methylbut-2-enyl) trisulfide (3-M-DBTS), and dihexenyl trisulfide (DHTS), induce cell apoptosis of PC-3 cells in a dose- and time-dependent manner through increasing the Bax/Bcl-2 ratio and activation of procaspase-3 [62].

Administration of DATS also significantly inhibits the progression of prostate carcinoma in transgenic adenocarcinoma of mouse prostate (TRAMP) mice and reduces the growth of PC-3 xenografts in athymic mice [63, 64]. The TRAMP mice are a well-known model for studying human prostate cancer, because they share many features important in human prostate cancer progression, including metastasis to distant sites, progression to androgen independence, and neuroendocrine differentiation [63]. Kim et al. also observed that the incidence of poorly differentiated prostate cancer is reduced by about 34–41% in the dorsolateral prostate of DATS-treated TRAMP mice in comparison with controls [59]. In another mouse model, DATS induces apoptosis and inhibits tumor cell proliferation, metastasis, and angiogenesis in BALB/c nude mice orthotopically transplanted with PC-3 prostate carcinoma compared with the control group [65].

### 5.4. SFN

SFN (Figure 2), a major isothiocyanate, is especially abundant in broccoli and broccoli sprouts. SFN has been widely demonstrated to induce prostate cancer cell apoptosis and reduce the growth of prostate cancer in animal models [66, 67]. H_2_S is able to mediate the antiproliferative role of SFN on prostate cancer cells through the activation of p38 mitogen-activated protein kinases (MAPK) and c-Jun N-terminal kinase (JNK) [19]. We previously observed that SFN acts as a slow-releasing H_2_S donor supported by several findings. Firstly, when SFN was added into cell culture medium with PC-3 cells, the concentration of H_2_S was doubled and lasted for at least 4 hours. Secondly, SFN released more H_2_S in the presence of liver homogenate, suggesting that SFN may liberate H_2_S under specific condition. SFN reacts with glutathione (GSH) to form a GSH conjugate in the mercapturic acid pathway, so it is highly possible that the existence of PC-3 cells or liver homogenates may provide enzymes to facilitate SFN binding with GSH for H_2_S liberation. Thirdly, halting of H_2_S production by methemoglobin or oxidized glutathione (two H_2_S scavengers) abolished SFN-stimulated MAPK activities and reversed the inhibitory role of SFN on PC-3 growth. Although SFN is well known to suppress prostate cancer in various animal models, recent phase II study reported that the treatment of prostate cancer patients with SFN-rich broccoli sprout extracts did not affect PSA level [68]. Further studies with higher doses of SFN-rich broccoli sprout extracts may be warranted to clarify the role of SFN as a prevention agent for prostate cancer.

## 6. H_**2**_S Interaction with Androgen Receptor

Androgen is essential for normal prostate physiology and plays a key role in either the initiation or progression of prostate cancer [69]. Androgen receptors (AR) can be activated by androgenic hormones and regulate the development of prostate cancer, as well as its transition to castration resistance state, and continued reliance on AR signaling is a hallmark of prostate cancer progression. The development of potential cancer chemopreventive and therapeutic agents to suppress AR signaling is highly desirable for clinical treatment on prostate cancer. Zhao et al. recently found that H_2_S suppresses AR transactivation but had no effect on AR protection expression, as evidenced by decreased AR binding with androgen responsive element (ARE) present in the promoter region of AR target genes. In addition, H_2_S lowers ARE luciferase activity [18]. Further studies demonstrated that H_2_S posttranslationally modifies AR proteins through* S*-sulfhydration. Both cysteine-611 and cysteine-614 present in the second zinc finger motif of DNA binding domain (DBD) are the target for H_2_S* S*-sulfhydration or AR protein, because mutation of these two cysteine residues completely abrogated* S*-sulfhydration of AR and AR dimer formation. It is predicted that the interaction of H_2_S with both cysteine-611 and cysteine-614 in AR-DBD alters local structure and leads to abnormal AR dimerization and DNA binding ability.

Another study showed that sulfide feeding of* nude* mice inoculated with human prostate cancer cells significantly decreases the expression of AR and its downstream genes PSA and NKX3.1, indicating that downregulation of the AR signaling pathway contributed to the inhibitory effects of sulfur on prostate cancer growth [43]. DATS is also shown to suppress AR function in prostate cancer cells. DATS incubation with prostate cancer cells (LNCaP, C4-2, and TRAMP-C1) decreases the protein expression of AR following the suppression of intracellular and secreted levels of PSA. Further studies showed that oligosulfide derived from DATS decreases AR promoter activity and AR mRNA level. DATS treatment inhibited synthetic androgen- (R1881-) stimulated nuclear translocation of AR in LNCaP/C4-2 cells. Interestingly, DATS treatment also caused a concentration-dependent decrease in phosphorylation of AR in LNCaP and C4-2 cells.* In vivo* data showed that oral gavage of DATS to TRAMP mice markedly inhibited AR protein level [70]. In contrast, DATS-mediated decrease in AR protein expression is insignificant in the normal prostate, suggesting DATS is unlikely to interfere with AR function in the normal prostate. Another H_2_S-releasing donor, SFN, also suppressed the expression of AR protein by inhibiting the cytoplasmic protein deacetylase HDAC6 in prostate cancer cells [71, 72].

## 7. Prospective

The realization of and interest in the functional importance of H_2_S in preventing cancer are growing. Despite the inconsistent and inconclusive findings in the field of H_2_S research, it appears that there is no doubt in the application of H_2_S in regulating numerous physiological and pathological conditions. Accurate determination of H_2_S levels in the circulation and tissues is challenging, but it is indispensable for further analyzing the levels of H_2_S and its metabolites in prostate cancer patients. Most of H_2_S donors extensively used in the present studies are of limited therapeutic value, due to the weakness of rapid release, instability, volatility, lack of specificity, and so forth. These limitations damper the enthusiasm for their further use as pharmaceutical drugs. Design and development of safer, controllable, and efficient H_2_S-based drugs to be locally delivered to prostate tissue are highly expected. Deciphering the molecular targets of H_2_S in prostate cancer progression at different stages will help us move forward to specific therapeutic applications. Despite the involvement of CSE/H_2_S system in AR signaling, their interactions in tumor development in both animal models and human prostate cancer patients remain to be elucidated. As more promising discoveries regarding H_2_S functions in prostate cancer rise to the surface, we expect more translation of the emerging roles of H_2_S in prostate cancer into human diagnostic and therapeutic approaches to evolve in the near future.

## Abbreviations Used

AR: Androgen receptor
ARE: Androgen responsive element
CBS: Cystathionine beta-synthase
CSE: Cystathionine gamma-lyase
DADS: Diallyl disulfide
DATS: Diallyl trisulfide
DBD: DNA binding domain
GSH: Glutathione
H_2_S: Hydrogen sulfide
MPST: 3-Mercaptopyruvate sulfurtransferase
NaHS: Sodium hydrosulfide
NH_3_: Ammonia
NO: Nitric oxide
PSA: Prostate-specific antigen
ROS: Reactive oxygen species
SFN: Sulforaphane
TRAMP: Transgenic adenocarcinoma of mouse prostate mice
TST: Thiosulfate: cyanide sulfurtransferase.
